# Predicting Risk for Death from MRSA Bacteremia^1^

**DOI:** 10.3201/eid1807.101371

**Published:** 2012-07

**Authors:** Mina Pastagia, Lawrence C. Kleinman, Eliesel G. Lacerda de la Cruz, Stephen G. Jenkins

**Affiliations:** The Rockefeller University, New York, New York, USA (M. Pastagia);; Mount Sinai School of Medicine, New York (L.C. Kleinman, E.G. Lacerda de la Cruz);; and Weill Cornell School of Medicine, New York (S.G. Jenkins)

**Keywords:** MRSA, bacteremia, heteroresistant *Staphylococcus aureus*, hVISA, vancomycin intermediate *Staphylococcus aureus*, VISA, mortality, death, bacteria, antimicrobial resistance

## Abstract

Methicillin-resistant *Staphylococcus aureus* (MRSA) bacteremia is often fatal. To determine predictors of risk for death, we conducted a retrospective cohort study. We examined 699 episodes of MRSA bacteremia involving 603 patients admitted to an academic medical center in New York City during 2002–2007. Data came from chart reviews, hospital databases, and recultured frozen MRSA specimens. Among the 699 episodes, 55 were caused by vancomycin–intermediate resistant *S. aureus* strains, 55 by heteroresistant vancomycin-intermediate *S. aureus* strains*,* and 589 by non–vancomycin-resistant strains; 190 (31.5%) patients died. We used regression risk analysis to quantify the association between clinical correlates and death. We found that older age, residence in a nursing home, severe bacteremia, and organ impairment were independently associated with increased risk for death; consultation with an infectious disease specialist was associated with lower risk for death; and MRSA strain types were not associated with risk for death.

## Abstract 2

Methicillin-resistant Staphylococcus aureus (MRSA) in the bloodstream is often fatal. Vancomycin is the most frequently prescribed drug for treatment of MRSA infections with demonstrated efficacy. Recently, however, some MRSA infections have not been responding to vancomycin, even those caused by strains considered susceptible. To provide optimal treatment and avoid vancomycin resistance, therapy should be tailored, especially for patients at highest risk for death. But who are these patients? A study that looked back at medical records and 699 frozen isolates found that risk for death from MRSA infection was highest among certain populations, including the elderly, nursing home residents, patients with severe sepsis, and patients with liver or kidney disease. Risk for death was not affected by the type of MRSA strain (vancomycin susceptible, heteroresistant, or intermediate resistant). Risk was lower among those who had consulted an infectious disease specialist. Thus, when choosing treatment for patients with MRSA infection, it is crucial to look at patient risk factors, not just MRSA strain type. For those at high risk, consultation with an infectious disease specialist is recommended.

Methicillin-resistant *Staphylococcus aureus* (MRSA) is a worldwide concern; it colonizes and infects patients in the hospital and in the community (*1*). For the past 50 years in the United States, the standard therapy has been vancomycin. Recent vancomycin treatment failures have raised questions regarding optimal treatment (*2*). Although new antimicrobial drugs (e.g., linezolid, daptomycin, tigecycline) have been developed, none has been consistently superior to vancomycin for the treatment of MRSA (*3**,**4*), and MRSA resistance rapidly develops for many new drugs (*5**,**6*). Some studies have suggested MIC creep (increasing vancomycin MICs against MRSA over time), but others have not (*7**,**8*). In 2006, the upper limit of vancomycin susceptibility for *S. aureus* was redefined, lowered from 4 µg/mL to 2 µg/mL, first by the Clinical and Laboratory Standards Institute and soon thereafter by the US Food and Drug Administration and the European Committee on Antimicrobial Susceptibility (*9*).

Vancomycin treatment failures for MRSA occur even when MICs are within the range considered susceptible, especially 1–2 µg/mL (*10**–**13*). Among high-risk bacteremic patients, Sakoulas et al. documented treatment failure rates of 44% when vancomycin MICs were <0.5 µg/mL and of 90% when vancomycin MICs were 1–2 µg/mL (p = 0.01) (*10*). Hidayat et al. found that mortality rates were higher for patients infected with strains with higher vancomycin MICs (*11*)

Some apparently susceptible strains of MRSA might actually be heteroresistant vancomycin-intermediate *S. aureus* (hVISA) strains. That is, although the hVISA isolates seem to be susceptible to vancomycin according to conventional testing, the isolates contain subpopulations of colonies resistant to vancomycin. Testing for hVISA has not been standardized and is not routinely undertaken. hVISA strains are more common in strains with higher vancomycin MICs (*14**,**15*). hVISA might contribute to worse clinical outcomes, but this possibility has not been convincingly confirmed by large studies.

To determine predictors of risk for death among patients with MRSA bacteremia, we conducted a retrospective study that compared demographic and clinical characteristics of adult patients with MRSA bacteremia. MRSA strains from these patients were vancomycin susceptible, VISA, and hVISA. We analyzed a 5-year trend of vancomycin MICs among adult patients with MRSA bacteremia. We also analyzed the associations between host factors, organism factors, and death versus survival, and quantifed the marginal contribution of key factors to risk for death.

## Methods

Our retrospective study was conducted in New York, New York, USA, at Mount Sinai Medical Center, a 1,171-bed tertiary-care academic center that serves a diverse ethnic and medical population. We studied 699 episodes of blood infection from 603 patients who had had MRSA bacteremia during 2002–2007. This study was approved by the institutional review board of the Mount Sinai School of Medicine.

### Laboratory Specimens

At Mount Sinai Medical Center, MRSA organisms identified from blood culture are routinely stored frozen at −70°C. We retrieved frozen blood culture isolates (previously not thawed or subcultured) for all adult patients hospitalized with MRSA bacteremia from January 2002 through May 2007. We excluded episodes of polymicrobial bacteremia if MRSA was isolated in a single blood culture bottle or if the patient received inappropriate empirical treatment for the co-pathogen. We included in our analysis the first organism isolated from blood culture during any episode of MRSA bacteremia.

A computerized data system identified 748 eligible isolates, among which we were able to retrieve 699 (93.4%). These isolates had originally been tested for drug susceptibility by use of an automated instrument, the Microscan (Siemens Healthcare, Sacramento, CA, USA); for all isolates, the vancomycin MICs were <2 µg/mL; during 2002–2007, we used the Positive Breakpoint Combo 20 (Siemens Healthcare), which might not accurately detect VISA isolates (*16*). We sent some isolates—8 (15%) VISA strains, 88 (15%) non-VISA strains, and 10 VISA control strains (from the Network on Antimicrobial Resistance in *Staphylococcus aureus*, www.narsa.net)—to an outside laboratory for blinded testing by using the Vitek 2 (bioMérieux, Durham, NC, USA) with the AST-GP-67 card, and we sent 8 (15%) hVISA isolates to an outside laboratory for retesting by using time-killing profiles. No discrepancies were noted.

Retesting of isolates was performed with no access (blinded) to clinical data. Mueller-Hinton agar plates (study and control strains) were inoculated with 0.5 McFarland inoculum (10^8^ CFU/mL), and antimicrobial drug susceptibility to vancomycin was assessed by using Etest (AB Biodisk, Solna, Sweden), which has excellent sensitivity and specificity for this purpose (*17*). Isolates for which vancomycin MICs were >1 µg/mL (95.1%) were tested for the presence of hVISA by using vancomycin and teicoplanin Etest strips (the Etest macromethod). In brief, study and control inocula equivalent to 2.0 McFarland turbidity standard were plated on brain–heart infusion and Mueller-Hinton agars and incubated at 35°C–37°C for 24 and 48 hours, respectively. Isolates were interpreted as being hVISA strains when vancomycin and teicoplanin MICs were >8 µg/mL or teicoplanin-only MIC was >12 µg/mL (*16*). Quality control testing was performed weekly by using American Type Culture Collection (Manassas, VA, USA) organisms 29213, 29212, 700698 (hVISA), and 700699 (VISA) per Clinical and Laboratory Standards Institute guidelines (*9*). Actual Etest values were used for MIC_50_ (the value below which 50% of MIC values for MRSA isolates tested fell), MIC_90_ (the value below which 90% of MICs fell), and geometric mean MIC calculations.

### Chart Review

We abstracted electronic and paper charts for each of the 603 patients corresponding to the 699 isolates; 65 of these patients were hospitalized >2 times for MRSA bacteremia. Chart abstraction was performed by 2 independent reviewers with no access (blinded) to laboratory data. Each reviewer separately examined 10% of charts; the κ statistic for coding of exemplar key variables was 0.87, indicating excellent agreement (*18*). Each new hospital admission was categorized as a new episode of bacteremia; the first positive blood culture was used as the index infection. We abstracted patient information regarding demographics, concurrent illnesses, patient’s residence before hospitalization (facility vs. community), bacteremia severity, and previous health care exposures.

### Definitions

We categorized each MRSA infection into 1 of 3 groups according to Etest result for vancomycin MICs as follows: VISA (MIC 4–12 µg/mL), hVISA, or non–VISA/hVISA MRSA (MIC <2 µg/mL). We also assessed MIC_50_ and MIC_90_ for vancomycin.

We defined the number of days to clearance of bacteremia as the date of first positive MRSA culture subtracted from the date of first negative culture for all patients for whom this information was available. Episodes of bacteremia ended on the date of death or on the date of the first negative blood culture that was not followed by a positive culture within 7 days. We did not study subsequent episodes during the same hospitalization. Vancomycin trough levels were measured after 3 doses of vancomycin; if multiple levels were measured, the modal level was used and classified as either >15 µg/mL or <15 µg/mL. We defined prior vancomycin exposure as receipt of >3 doses of vancomycin at least 7 days and <12 months before MRSA bacteremia. Empirically prescribed therapy was defined as appropriate if MRSA were susceptible to the antimicrobial drug used (according to in vitro susceptibility testing) and if therapy was started within 48 hours of the blood culture result. History of MRSA infection was defined as hospitalization with MRSA during the prior 12 months. We defined an episode as community associated if the patient was bacteremic within 48 hours of hospitalization and lacked health care–associated risk factors such as dialysis, nursing home residence, or history of MRSA infection. We divided health care–associated MRSA cases into community onset or hospital onset. Cases were health care–associated community onset if the patient had such risk factors and was found to be bacteremic within 48 hours of hospital admission; cases were of health care–associated hospital onset if the bacteremia occurred after 48 hours of hospitalization, consistent with the schema of Klevens et al. (*19*).

We defined renal insufficiency as serum creatinine level >2 mg/dL or glomerular filtration rate <50 mL/min/1.73 m^2^ according to the Cockcroft-Gault equation. The source of the bacteremia was determined by a combination of positive MRSA culture growth from a site other than blood, radiologic evidence, or an attending physician’s statement in the medical record. We used the Duke criteria to define endocarditis (*20*). Severity of bacteremia at onset of infection was determined by use of vasopressors, elevation of serum creatinine levels from baseline (renal insufficiency), and admission to an intensive care unit after positive MRSA blood culture result. The major patient outcome measure was 90-day all-cause mortality rate. Mortality rate was determined by calculating deaths from the date of positive MRSA blood culture result up to 90 days while hospitalized, divided by the number of patients in the study (n = 603). Data regarding death after hospital discharge were not available for analysis. MRSA-attributable deaths were not included because of the difficulty in assessing exact cause of death.

### Statistical Analysis

We used SAS version 9.1 (SAS Institute, Cary, NC, USA) for statistical analyses (*21*). We used standard methods to describe univariate data and to calculate *t*-tests, and we used χ^2^ for bivariate associations. We used generalized linear models (SAS Proc GLM) to assess associations between the 3 groups of MRSA infection and quantitative variables. We conducted multivariable analyses by using logistic regression (SAS Proc Logistic). We developed models for 3 variables: infection with VISA, infection with hVISA, and death. Model building was guided first by conceptual models of likely effect and informed by our bivariate analysis results. We assessed correlation coefficients between pairs of potential predictor variables by using appropriate parametric or nonparametric methods and included only 1 of any pair of variables with an r^2^ of >0.25 in any model. Guided by the rule for stability of estimates established by Peduzzi et al., we limited the total number of predictor variables in any model (*22*). The significance of the models (Table 1, Table 2) are demonstrated by likelihood ratios, Wald <0.0001. When choosing between similar or comparable models, we selected the model associated with the smallest Akaike information criterion (<582); for example, in our analysis of predictors of risk for death, we rejected variables indicating HIV, malignancy, transplant, recent surgery, and presence of a medical device because they increased the Akaike information criterion. The predictive and discriminative performance of our models is shown in Tables 1 and 2. Our model predicting death has a c-score of 0.872; among 99,198 pairs, 87.1% were concordant, 12.7% were discordant, and 0.2% had the same scores. Although we also present the more familiar adjusted odds ratios, our primary measures of impact are adjusted risk measures (adjusted risk ratio and adjusted risk difference), which we derived from regression risk analysis, an enhancement over the usual presentation of logistic regression (*23*).

**Table 1:** Multivariable analysis of risk factors for VISA and hVISA infections, New York, New York, USA, 2002–2007*

Risk factor and MRSA strain	Odds ratio	Adjusted risk ratio (95% CI)	Adjusted risk difference (95% CI)†
Age‡			
VISA	0.93	0.82 (0.65 to 1.12)	–0.020 (–0.080 to 0.007)
hVISA	0.93	0.96 (0.73 to 1.35)	–0.003 (−0.050 to 0.013)
Race/ethnicity			
Black			
VISA	0.90	0.76 (0.35 to 1.57)	–0.02 (–0.07 to 0.04)
hVISA	0.90	1.03 (0.44 to 2.13)	0.002 (−0.050 to 0.070)
Hispanic			
VISA	0.89	0.87 (0.36 to 1.88)	–0.01 (–0.06 to 0.05)
hVISA	0.46	0.62 (0.19 to 1.37)	−0.03 (−0.08 to 0.03)
Asian			
VISA	1.97	1.79 (0.77 to 3.34)	0.06 (−0.02 to 0.15)
hVISA	1.58	1.65 (0.66 to 3.21)	0.05 (−0.03 to 0.14)
Concurrent condition			
Diabetes			
VISA	1.42	1.44 (0.78 to 2.59)	0.03 (−0.02 to 0.08)
hVISA	0.69	0.87 (0.48 to 1.48)	−0.01 (−0.05 to 0.03)
Chronic hemodialysis			
VISA	1.25	1.23 (0.56 to 2.57)	0.02 (−0.04 to 0.09)
hVISA	1.32	1.05 (0.48 to 2.03)	0.004 (−0.040 to 0.060)
HIV			
VISA	0.50	0.45 (0.09 to 1.06)	–0.05 (−0.090 to 0.004)
hVISA	0.30	0.27 (0.06 to 1.38)	−0.060 (−0.100 to −0.008)
Liver cirrhosis			
VISA	2.38	3.43 (2.02 to 6.00)	0.14 (0.06 to 0.23)
hVISA	2.55	2.11 (1.06 to 3.87)	0.080 (0.005 to 0.170)
Malignancy			
VISA	2.02	1.96 (1.07 to 3.31)	0.070 (0.005 to 0.130)
hVISA	1.37	1.64 (0.87 to 3.07)	0.04 (−0.01 to 0.11)
Other			
Nursing home residence			
VISA	1.62	1.83 (0.88 to 3.30)	0.060 (–0.009 to 0.130)
hVISA	1.12	0.94 (0.36 to 1.80)	–0.005 (–0.060 to 0.050)
Surgical procedure§			
VISA	0.50	0.41 (0.20 to 0.76)	–0.06 (–0.10 to –0.02)
hVISA	0.79	0.62 (0.32 to 1.02)	–0.040 (–0.080 to 0.002)
Prior receipt of vancomycin			
VISA	1.87	2.09 (1.25 to 3.67)	0.06 (0.02 to 0.10)
hVISA	0.92	0.97 (0.56 to1.65)	−0.002 (−0.040 to 0.040)
Central venous catheter infection			
VISA	0.80	0.83 (0.50 to1.36)	–0.01 (–0.05 to 0.02)
hVISA	2.09	1.81 (1.13 to 3.10)	0.050 (0.009 to 0.090)

*Results from logistic regression with outcomes and covariates. VISA, vancomycin-intermediate *Staphylococcus aureus* strains, hVISA, heteroresistant vancomycin-intermediate *S. aureus* strains; MRSA, methicillin-resistant *S. aureus*. †Adjusted risk difference refers to the absolute difference in risk; for example, an adjusted risk difference of 0.10 signifies a 10% increased risk for hVISA, VISA, given that variable. ‡Measures are adjusted odds ratio for a 10-y difference and adjusted risk difference for effect of age 50–60 y. §Surgical procedures under general anesthesia within the past 3 months.

**Table 2:** Multivariable analysis of risk factors for 90-day all-cause deaths among 603 patients with MRSA bacteremia, New York, New York, USA, 2002–2007*

Risk factor	Odds ratio (95% CI)	Adjusted risk ratio (95% CI)	Adjusted risk difference† (95% CI)
Age‡	1.72 (1.29 to 2.30)	1.34 (1.12 to 1.65)	0.04 (0.03 to 0.05)
Race/ethnicity			
Black	0.71 (0.39 to 1.29)	0.85 (0.63 to 1.16)	–0.04 (–0.04 to –0.11)
Hispanic	0.85 (0.45 to 1.58)	0.93 (0.66 to 1.23)	–0.02 (–0.10 to 0.06)
Asian	1.83 (0.92 to 3.66)	1.30 (0.95 to 1.72)	0.08 (–0.01 to 0.18)
Concurrent condition			
Diabetes	0.50 (0.31 to 0.83)	0.73 (0.57 to 0.93)	–0.08 (–0.14 to –0.02)
Immunosuppressant use	0.83 (0.37 to 1.83)	0.92 (0.63 to 1.30)	–0.02 (–0.10 to 0.08)
Liver cirrhosis	2.18 (1.16 to 4.12)	1.40 (1.04 to 1.77)	0.10 (0.01 to 0.19)
Renal insufficiency	1.89 (1.18 to 3.01)	1.33 (1.05 to 1.70)	0.08 (0.01 to 0.14)
Other			
Infectious disease consultation	0.43 (0.26 to 0.69)	0.69 (0.57 to 0.86)	–0.11 (–0.16 to –0.04)
History of MRSA infection	0.77 (0.45 to 1.34)	0.89 (0.70 to 1.13)	–0.03 (–0.09 to 0.04)
Nursing home residence	3.08 (1.81 to 5.24)	1.62 (1.31 to 2.06)	0.15 (0.08 to 0.23)
Intensive care unit stay	1.71 (1.17 to 2.50)	1.29 (1.11 to 2.15)	0.07 (0.03 to 0.20)
Vasopressor use	15.44 (8.58 to 27.76)	3.67 (2.66 to 4.66)	0.48 (0.34 to 0.58)
Inappropriate antimicrobial drug therapy	1.38 (0.73 to 2.63)	1.15 (0.89 to 1.46)	0.04 (–0.03 to 0.12)
MRSA strain			
VISA	0.58 (0.24 to 1.38)	0.78 (0.49 to 1.19)	–0.06 (–0.15 to 0.05)
hVISA	1.23 (0.54 to 2.82)	1.10 (0.67 to 1.58)	0.03 (–0.09 to 0.16)
Infection source			
Pneumonia	1.77 (0.85 to 3.64)	1.28 (0.91 to 1.68)	0.07 (–0.02 to 0.17)
Vascular graft infection	0.21 (0.03 to 1.70)	0.460 (0.005 to 0.940)	–0.15 (–0.29 to –0.02)
Endocarditis	1.49 (0.80 to 2.79)	1.19 (0.87 to 1.51)	0.05 (–0.03 to 0.13)

*Results from logistic regression with outcomes and covariates. MRSA, methicillin-resistant *Staphylococcus aureus*. VISA, vancomycin-intermediate *S. aureus* strains, hVISA, heteroresistant vancomycin-intermediate *S. aureus* strains. †Adjusted risk difference refers to the absolute difference in risk; for example, an adjusted risk difference of 0.10 signifies a 10% increased risk for all-cause deaths given that variable. ‡Measures are adjusted odds ratio for a 10-y difference and adjusted risk difference for effect of age 50–60 y.

## Results

### Bacteria Characteristics

Each year during 2002–2006, the annual number of hospital admissions in this study were 117, 77, 147, 121, and 161, respectively; through May 31, 2007, another 76 patients were hospitalized (equivalent to 184 annual hospitalizations). The original testing of strains by Microscan did not detect VISA; repeat testing using the Vitek 2 detected 2 (25%) of 8 study strains and 2 (20%) of 10 control strains. The rate at which polymicrobial bacteremia met inclusion criteria was 3% (20 episodes). The Figure demonstrates the proportion of VISA, hVISA, and non–VISA/hVISA MRSA by year and the increase in mean vancomycin MICs during the study period. The vancomycin MICs for most (87%) isolates were 1–2 µg/mL. For VISA, MICs were as high as 12 µg/mL, although for 60%, MICs were 4 µg/mL. For 94% of hVISA strains, vancomycin MICs were 1.5–2.0 µg/mL.

**Figure:**
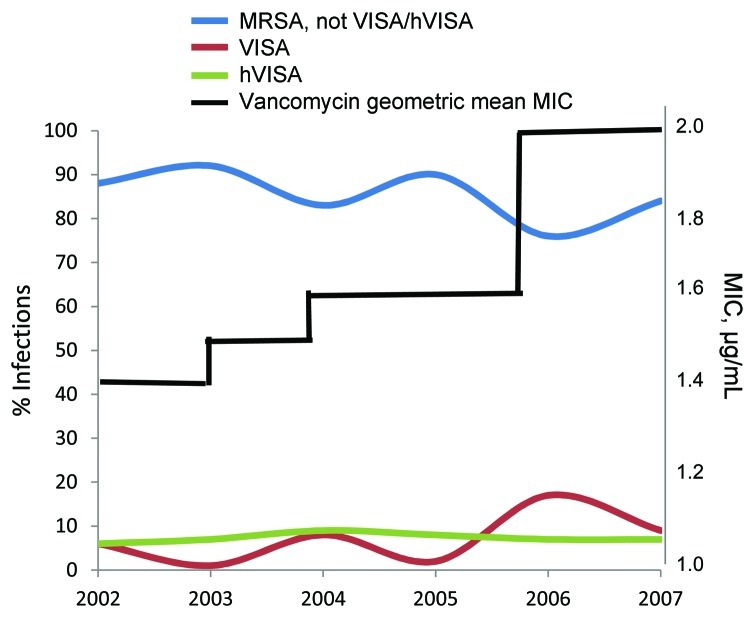
Trend of methicillin-resistant *Staphylococcus aureus* (MRSA) infection strain types, New York, New York, USA, 2002–2007. VISA, vancomycin-intermediate *S. aureus* strains, hVISA, heteroresistant vancomycin-intermediate *S. aureus* strains.

The geometric mean MIC of vancomycin was 1.7 µg/mL; modal MIC = 2 µg/mL. In 2002, the MIC_50_ of vancomycin was 1.5 µg/mL; by 2007, it was 2 µg/mL. The MIC_90_ was constant during the study period; vancomycin MIC_90_ was 4 µg/mL in 2002 and 2004–2007.

### Patient Characteristics

Key patient characteristics are shown in Table 3. Recent medical care seemed to be associated with type of strain. Nearly 40% of infections were health care–community associated, almost all the rest were hospital associated. Average length of stay for patients with all infection types was 32.9 days. Many cases of MRSA bacteremia were in patients with renal insufficiency and/or cardiovascular disease; >40% had recently had a surgical procedure. Many (43%) cases of bacteremia were secondary to central venous catheter infections. The 90-day all-cause mortality rate was 31.5% for the 603 patients; rate was 27.2% when all 699 episodes of bacteremia were considered.

**Table 3:** Clinical characteristics for patients with MRSA bacteremia by strain type, New York, New York, USA, 2002–2007*

Characteristic	VISA, n = 55	hVISA, n = 55	non–VISA/hVISA MRSA, n = 589	p value
Age, mean y ± SD†	58.7 ± 16.5	58.7 ± 16.5	63 ± 17.3	0.04
Length of stay, mean d ± SD	33.7 ± 41.4	30.9 ± 22.7	34 ± 41.9	0.49
Days to negative culture, mean ± SD	3.7 ± 2.8	3.6 ± 3.2	4.3 ± 4.8	0.98
Male sex	29 (52.7)	28 (50.9)	340 (57.7)	0.51
Race/ethnicity				
White	19 (34.5)	21 (38.2)	238 (40.4)	0.59
Black	15 (27.3)	14 (25.5)	160 (27.2)	0.96
Hispanic	12 (21.8)	7 (12.7)	123 (20.9)	0.34
Asian	9 (16.4)	10 (18.2)	65 (11.0)	0.17
General				
Hospitalization within 1 mo of MRSA infection	29 (52.7)	17 (30.9)	295 (50.1)	0.02
Prior MRSA infection	22 (40.0)	7 (12.7)	159 (27.0)	0.006
Prior vancomycin exposure	34 (61.8)	25 (45.5)	272 (46.2)	0.08
Vancomycin trough >15 µg/mL	34 (61.8)	25 (45.5)	387 (65.7)	0.0005
Inappropriate antimicrobial drug therapy	12 (21.8)	4 (7.3)	66 (11.2)	0.09
Infectious diseases consultation	34 (61.8)	23 (41.8)	404 (68.6)	0.0003
Health care–associated hospital infection	19 (34.5)	28 (50.9)	340 (57.7)	0.003
Health care–associated community infection	31 (56.4)	25 (45.5)	241 (40.9)	0.08
Concurrent conditions				
Renal insufficiency	35 (63.6)	29 (52.7)	309 (52.5)	0.48
Chronic hemodialysis	19 (34.5)	12 (21.8)	128 (21.7)	0.09
Diabetes mellitus	27 (49.1)	18 (32.7)	209 (35.5)	0.11
HIV	5 (9.1)	2 (3.6)	60 (10.2)	0.29
Cardiovascular disease	33 (60.0)	31 (56.4)	399 (67.7)	0.14
Malignancy	14 (25.5)	17 (30.9)	117 (19.9)	0.11
Transplant	7 (12.7)	11 (20.0)	46 (7.8)	0.007
Cirrhosis	22 (40.0)	14 (25.5)	78 (13.2)	<0.0001
Steroids	17 (30.9)	12 (21.8)	193 (32.8)	0.25
Surgery <3 mo before MRSA infection	14 (25.5)	19 (34.5)	271 (46.0)	0.005
Implanted device	8 (14.5)	7 (12.7)	151 (25.6)	0.02
Intensive care unit stay	23 (41.8)	26 (47.3)	273 (46.3)	0.96
Infection source				
Central venous catheter	27 (49.1)	33 (60.0)	242 (41.1)	0.04
Pneumonia	10 (18.2)	6 (10.9)	39 (6.6)	0.62
Endocarditis	7 (12.7)	3 (5.5)	81 (13.8)	0.22
Wound/skin or soft tissue	10 (18.2)	10 (18.2)	92 (15.6)	0.80
Bone/joint	6 (10.9)	1 (1.8)	65 (11.0)	0.10
Vascular graft	2 (3.6)	1 (1.8)	17 (2.9)	0.85
Death within 90 d of MRSA infection	15 (27.3)	14 (25.5)	161 (27.3)	0.38

*Values are no. (%) with a given variable per strain type unless otherwise indicated. MRSA, methicillin-resistant *Staphylococcus aureus;* VISA, vancomycin-intermediate *S. aureus* strains, hVISA, heteroresistant vancomycin-intermediate *S. aureus* strains. †N = 699.

### Treatment and Changes in Treatment Regimens

Among the 603 patients, 47% had been exposed to vancomycin and 60% of these had had prior MRSA infection. Prior vancomycin exposure was more likely for patients with VISA (62%, 95% CI 47.7%–65.3%) than for patients with hVISA (42%, 95% CI 29.4%–59%) or other MRSA strains (47%, 95% CI 41.3%–49.7%).

Among the 699 episodes of MRSA bacteremia, vancomycin was used to treat 566 (81%) episodes. Initial vancomycin treatment was switched to daptomycin or linezolid for 12% of MRSA (non-VISA, non-hVISA) infections and 15% of VISA infections. For the 699 episodes of bacteremia, mortality rates were 27.2% overall, 16% (95% CI 6.8%–24.8%) when antimicrobial drug treatment was changed, and 26% (95% CI 22.2%–29.8%) when not changed. Our data did not enable us to determine the extent to which switching, or not switching, antimicrobial drugs contributed to survival.

### Correlates of VISA and hVISA Infections

Multivariable analyses (Table 1) demonstrate associations between key clinical characteristics and VISA or hVISA infections. The adjusted risk difference represents the absolute difference in risk for that given characteristic, all else held equal. Cirrhosis of the liver and central venous catheter infections nearly doubled the risk for hVISA infection. Cirrhosis and active malignancy increased the absolute risk for VISA by 14% and 7%, respectively. History of vancomycin exposure within 1 year increased the risk for VISA (6%) but did not increase the risk for hVISA infection.

### Predictors of All-Cause Death

The effect of various clinical characteristics on risk for death within 90 days is summarized in Table 2. For patients with concomitant MRSA bacteremia, older age increased the risk of dying. Cirrhosis or renal insufficiency and having lived in a nursing home before hospitalization or having been admitted to an intensive care unit were each independently associated with death (after adjusting for covariates in the model); each increased the risk of dying by 7%–15%. Patients who required vasopressors had an absolute increase in risk for death of ≈50%, after covariates were adjusted for. Risk for death was independently associated with lower risk for death among those who had diabetes mellitus or who had had a vascular graft as the source of the infection. A consultation with an infectious diseases specialist decreased the risk for death by 11%. Neither a VISA nor hVISA strain was independently associated with all-cause death after covariates in the models were controlled for.

A subanalysis of vancomycin MICs for strains infecting patients who died in the hospital found that the mean MIC was 1.7 µg/mL. The current breakpoint of vancomycin susceptibility is 2 µg/mL.

## Discussion

The idealized model for the treatment of patients with infectious diseases incorporates the triad of host, organism, and drug. Organisms and drugs are more easily classified and hence more accessible for systematic study. Our study of the 5-year experience with MRSA infections in adults at a major New York City medical center illustrates why such a dyadic approach might be insufficient. For example, the MIC, which characterizes the major intersection between organism and drug, was overshadowed by a constellation of clinical factors when predicting risk for death. Vancomycin MICs from isolates from most persons who died indicated nominal susceptibility. Several other studies have shown vancomycin MIC to not be a predictor of death (*10**,**12**,**13*).

Unlike others, who considered concurrent conditions by using scales such as the Charlson Index (*15**,**24*), we investigated the association between specific patient characteristics, organisms, drugs, and outcomes. Not all concurrent conditions were alike in either magnitude or direction of effect. Regression risk analysis enabled us to identify the independent contribution of these factors in relative and absolute terms. We identified critical prognostic factors, including concurrent conditions (cirrhosis and renal insufficiency suggested a poorer prognosis; diabetes, a better one) and source of admission (nursing home residence suggested a poorer prognosis). We learned that strain type was not an independent negative prognostic factor. As one might expect, the use of vasopressors presaged an increased risk for death (adjusted risk difference = 48%).

Our findings can help clinicians estimate the risk that a patient with MRSA bacteremia will die. For example, an elderly patient with liver cirrhosis and MRSA bacteremia who lived in a nursing home before hospital admission would have an extremely poor prognosis. Conversely, an otherwise healthy patient with diabetes mellitus might have a better prognosis that could be improved even more by consultation with an infectious disease specialist. We note paradoxically that several of the positive predictive factors (such as diabetes and vascular graft infections) represent situations in which host barriers to infections might be impaired. We speculate that host, organism, and drug factors might all interact; an impaired host might become infected by a less aggressive organism that in turn is more susceptible to drugs. In this study, ≈30% of patients with skin and soft tissue infections had diabetes mellitus with varying levels of baseline glucose control. Thus, the source of infection and spectrum of disease might also affect risk for death. This and alternate hypotheses should be explored in future research.

There is controversy regarding the value of testing for hVISA (*25*). Although this article is unlikely to resolve that controversy, we can say that patients infected with these strains in our cohort probably did not have increased risk for death from all causes. Other reports suggest that a down-regulation of virulence might be associated with increased vancomycin resistance (*12**,**26*).

We observed trends in which vancomycin MICs crept upward over the 5 years of the study. As a corollary, the likelihood of VISA infections increased. The rate of hVISA infections during 2002–2007 was steady, around 8%, similar to that described in the literature (*27**,**28*). Prior exposure to vancomycin was a contributing factor for infection with VISA strains (*29**–**31*). Although we describe a vancomycin MIC creep, the MIC_90_ of vancomycin remained relatively stable over the 5-year period, perhaps hinting at why MIC did not independently predict death.

A recent study by Paul et al. found a significant increase in 30-day mortality rates for patients given incorrect therapy within 48 hours of blood culture (*32*). Schweizer et al. did not find an increase in hospital deaths among similar patients with *S. aureus* bacteremia (*33*). Our study was limited by our inability to assess deaths that occurred out of the hospital.

Consistent with mortality rates reported in the literature, ≈32% of our patients died (*34*). Considering the marginal impact of several independent risk factors, our innovative presentation of adjusted risk differences offers clinicians a quantifiable way to assess their patients’ risk for death. Although the numbers were too low to analyze with multivariable models, we note the trend toward improved prognosis among those for whom antimicrobial drug therapy was switched and recognize that because switches are likely for patients who are not doing well clinically, that the most apparent bias is against such a finding. Thus, our findings hint at potential benefit for prescribing alternative drugs if patients are not improving. Two recent studies have shown that consultations with an infectious diseases specialist lower the risk for death from *S. aureus* bacteremia (*35**,**36*).

This study is limited by its retrospective design and single-center setting. MICs derived by using Etest might be higher than those derived by microdilution (*37*). hVISA strains were identified by using a method with demonstrably high sensitivity and specificity and not by using the standard method (*10**,**38**,**39*). We did not test for hVISA on the 5% of isolates for which vancomycin MIC was <1 µg/mL; other studies have noted few or no hVISA in this MIC group (*10**,**24**,**25**,**27*). The accuracy of timing of vancomycin trough levels was limited. Daily bacterial cultures were not always conducted, decreasing the data points for clearance of bacteremia. Misclassification error is possible because we reviewed records available at Mount Sinai Medical Center only. Almost all study patients had sufficient contact with the health care system that not many infections were classified as community acquired, limiting our ability to generalize to those infections. As noted, we were limited by our inability to link to death data outside of the hospital records.

Until now, the major focus on active MRSA infections has been on the organism and its susceptibility to the drug. Although decreased vancomycin susceptibility has resulted in prolonged bacteremia and treatment failure in several studies (*15**,**24*), our findings suggest that incorporating the context, the host, and the environment is similarly useful. Our study emphasizes that after a diagnosis of MRSA bacteremia is made, it is crucial to determine patient risk factors and not just the vancomycin MIC for the infecting strain. The consequences of MRSA bacteremia are clear—many patients will die or experience a decline from their baseline clinical condition. The adjusted risk difference enables clinicians to use a targeted approach, directed toward patients with the highest risk for death—i.e., the elderly, patients with liver cirrhosis, patients with renal insufficiency, and patients from nursing homes. These patients should be treated carefully and should possibly receive a consult from an infectious diseases specialist. What remains unclear is whether patients with increased risk for death should be treated with antimicrobial drugs other than vancomycin.
